# Bio-Based Epoxy Resins Based on Linseed Oil Cured with Naturally Occurring Acids

**DOI:** 10.3390/polym11091409

**Published:** 2019-08-28

**Authors:** Kerstin Thiele, Nicole Eversmann, Andreas Krombholz, Daniela Pufky-Heinrich

**Affiliations:** 1 Fraunhofer Center for Chemical-Biotechnological Processes CBP, Gate 12, Building 1251, 06237 Leuna, Germany; 2 Fraunhofer Institute for Microstructure of Materials and Systems IMWS, Walter-Hülse-Straße 1, 06120 Halle, Germany

**Keywords:** epoxidized linseed oil, epoxy resin, methyltetrahydrophthalic anhydride, pyromellitic dianhydride, maleic acid, citric acid, oxalic acid

## Abstract

Structural properties of resins based on epoxidized linseed oil (ELO) were investigated in reference to varying amounts of the hardener components methyltetrahydrophthalic anhydride (MTHPA), pyromellitic dianhydride (PMDA) and maleic acid (MA). This includes gel time and the Shore A and D hardness. The shortest gel time of 0.9 min and the highest Shore A and D hardness of 85 and 34 were found at a n_MTHPA_/n_PMDA_/n_MA_ molar ratio of 8/1/8. To study the effect of the ELO mass on gel time and hardness, different masses of ELO (8, 10, 12, 14 and 16 g) were used, keeping the amount of the hardener system (4 g) (MTHPA, PMDA and MA) constant. With increased ELO mass, gel time increased while the Shore A and D hardness of the samples did not differ when up to 14 g ELO was applied. Substitution of petrol-based PMDA with biogenic compounds, specifically oxalic acid and citric acid, resulted in new bio-based epoxy resins with shorter gel times while maintaining hardness.

## 1. Introduction

The substitution of petrol-based chemicals with compounds from sustainable resources is becoming more and more attractive due to the price increase of fossil reserves as well as environmental and economic advantages. Epoxy resins based on diglycidyl ether of bisphenol A (DGEBA) are an important and broadly utilized material class. They have been used for example as structural adhesives, in anticorrosive coating or for conductor boards because of their mechanical and electrical properties as well as heat and chemical resistance [1,2]. However, as DGEBA is harmful to health [3,4], the development of new bio-based polymers has come to the forefront of research [5,6,7]. 

As they are cost-effective, ecofriendly and relatively abundant, vegetable oils are a preferable source of polymeric resins [8,9,10,11]. As an epoxidized derivative, linseed oil from flax, for example, can be used as a good alternative due its property of being the most highly molecularly-unsaturated (6.6 double bonds per triglyceride chain) of all plant oils. It is a valuable crop and every part of the plant has its own specific economic importance. In the last report in 2009, Canada had the highest share in the production of linseed (44%), followed by China (15%), Europe (12%) and the USA (9%) [12]. It is used as an industrial and edible oil and for fiber-producing crop. There are also medical applications due to it being rich in fat (41%), protein (20%) and dietary fiber (28%) [12]. It is mainly composed of triglyceride molecules, which are composed of three fatty acids bound by a glycerol center through ester linkages. The choice of the right curing agent is very important to achieve sufficient mechanical and thermal properties of the epoxy resins. In industrial applications, amines are a typically used curing agent. However, due to their toxicity, alternative curing agents are necessary. For example, Sahoo et al. [13] used cardanol-derived phenalkamine as a bio-based cross-linker.

**Figure 1:**
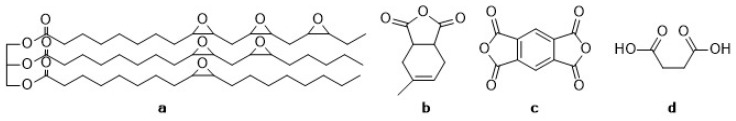
Structural formulae of (**a**) the epoxidized linseed oil (ELO) and the compounds of the hardener system (**b**) methyltetrahydrophthalic anhydride (MTHP), (**c**) pyromellitic dianhydride (PMDA) and (**d**) maleic acid (MA).

In the literature, methyltetrahydrophthalic anhydride (MTHPA) [14,15] and pyromellitic dianhydride (PMDA) [16] have been studied as hardeners for epoxidized linseed oil (ELO). In this study, blends of MTHPA, PMDA and maleic acid (MA) were used as a hardener system and the gel time with ELO was investigated (see structural formulae in Figure 1). The Shore A and D values detected in the epoxy materials provided information about their hardness. To increase the biogenic character of the epoxy resins, petrol-based PDMA of the hardener system was to be substituted with bio-renewable chemicals like oxalic acid (OA), fumaric acid (FA), succinic acid (SA), malic acid (HAS), tartaric acid (TA), itaconic acid (IA) and citric acid (CA).

## 2. Materials and Methods

### 2.1. Materials

Citric acid monohydrate (Roth, Karlsruhe, Germany, ≥99.5%), fumaric acid (Merck, Darmstadt, Germany, >98%), itaconic acid (Merck, ≥99%) linseed oil epoxy (ELO, EPOL-L Traditem, Hilden, Germany), maleic acid (TCl, Zwijndrecht, Belgium, >99%), malic acid (Merck, ≥98%), methyltetrahydrophthalic anhydride (TCl, >80%), oxalic acid dihydrate (VWR, Darmstadt, Germany, ≥99%), pyromellitic dianhydride (TCl, >99%), succinic acid (Merck, ≥99%) and tartaric acid (Aldrich, Steinheim, Germany, ≥99.5%) were used as received.

### 2.2. Sample Preparation

To optimize the curing behavior of the resins and thereby maximize hardness and minimize gel time, the relative molar ratio of the hardener was varied (Table 1). The sample names include the abbreviation “PD” for pyromellitic dianhydride since other chemicals will later replace this hardener compound. These samples will be described with the abbreviation of the respective acid: oxalic acid (OA), fumaric acid (FA), succinic acid (SA), malic acid (HSA), tartaric acid (TA), itaconic acid (IA) and citric acid (CA). The number that follows refers to the molar ratios in integers. MTHPA is the only liquid component in the hardener system, the other components are powdery and poorly soluble within the liquid component. 

For synthesis of resin, the hardener mixtures (4 g) were grinded for 5 minutes in a porcelain mortar to produce a very fine dispersion. Some of the dispersions with higher amounts of MTHPA were opaque liquids; lower amounts would lead to pasty or even powdery mixtures. ELO (12 g) was added and mixed in the mortar with the hardener, until no particles were visible. The exothermic reaction of the hardener with ELO started immediately at room temperature. For the measurement of Shore A and D hardness, the mixtures were poured into small polypropylene cups. The samples were cured at 23 °C and at a relative humidity of 50% for one week and then removed from the mold before measurement.

In order to investigate gel time, after being quickly mixed, the material was filled into a plastic syringe, placed into the cavities of the b-time plate and measured at 80 °C. 

### 2.3. Characterization

Determination of gel-time occurred according to DIN EN ISO 8987:2005-12 [17]. For each mixture. Five samples of ca. 500 mg fresh mixed liquid resin were measured. The plate was heated at 80 °C and then ca. 500 mg of the resin was placed by syringe into the cavities of the plate while time measurement was simultaneously started. The resin was stirred with a wooden stick, which was frequently lifted to test if the resin was still producing filaments. When the resin began rubber-like tearing, time measurement was stopped. 

Shore A and D hardness was measured in cured samples. The curing took place at 23 °C and a relative humidity of 50% for one week. The measurement occurred in accordance with the appropriate test method [18].

## 3. Results and Discussion

### 3.1. Prepared Epoxy Resins with Varying Composition of the Hardener System and ELO

**Figure 2:**
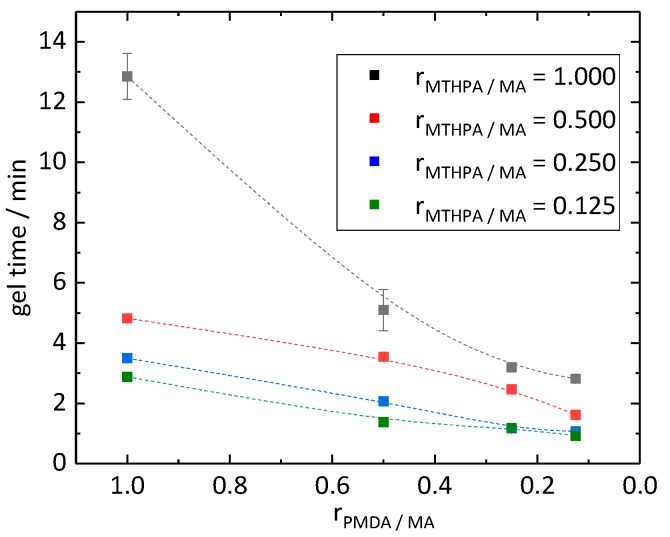
Gel time of epoxide resins in reference to the molar ratios r_PMDA/MA_ and r_MTHPA/MA_.

Curing of the epoxidized linseed oil (ELO) with a hardener depends on the nature of the hardener as well as on the stoichiometry of the epoxy groups and reactive groups of the hardener. The epoxy group can react with both an alcohol and a carboxylic group. The formed alcohol can further react with a carboxylic anhydride. Due to these simultaneous reactions, a balanced reaction equation of the curing agent with the ELO to the resin is not feasible and a structural formula of the epoxy resin is not predictable. With a stoichiometric ratio of around 2.5 of epoxy to reactive groups, the excess epoxy was used in accordance with Boquillon et al. [19]. The gel time test showed that when decreasing molar ratios r_PMDA/MA_ and r_MTHPA/MA_ from 1.000 to 0.125, the curing time decreased from 12.9 to 0.9 min (Figure 2). The highest decrease of gel time, from 12.9 to 5.1 min, took place by decreasing the molar ratio r_PMDA/MA_ from 1.000 to 0.500 while keeping a constant molar ratio r_MTHPA/MA_ of 1.000. The curves of the materials with molar ratio r_MTHPA/MA_ of 0.500, 0.250 and 0.125 proceeded similarly to each other, only shifting by circa 0.5 min and 1.0 min, respectively, along the y (gel time) axis. MTHPA remained at room temperature in its liquid state. The other components of the curing agent were present in solid state. Due to the different states of matter, the mixtures of the curing agents showed different states as well. The mixture of the curing agent of the PD418 sample was in a pasty state, while with a lower molar ratio _rMTHPA/MA_ of 0.250 (sample PD144), a powder was present. Compared to them, the hardener systems with a molar ratio r_MTHPA/MA_ higher than 0.250 were in liquid state, which were easier to apply. The PD418 sample (r_MTHPA/MA_ = 0.500 and r_PMDA/MA_ = 0.125) was the most promising sample due to its gel time of 1.6 min and its liquid state, which was easier to use and handle compared to the pasty samples. 

**Figure 3:**
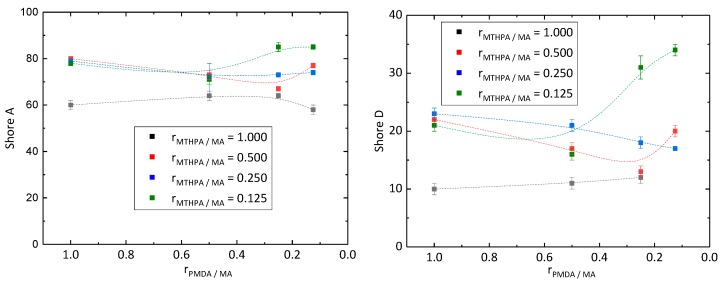
Shore A (**left**) and D (**right**) hardness of epoxy resins in reference to the molar ratio r_PMDA/MA_ and r_MTHPA/MA_.

Hardness of the synthesized epoxy resins was examined by Shore A and Shore D hardness under normal climate conditions, due to the requirements for its application as a coating (Figure 3). Epoxy resins with Shore A values up to 20 are classified as very soft, values between 20 and 40 as medium hard, and values above 40 as very hard [20]. All samples showed Shore A values higher than 40 meaning they exhibited a high level of hardness. Samples PD128 (r_MTHPA/MA_ = 0.125 and r_PMDA/MA_ = 0.250) and PD118 (r_MTHPA/MA_ = 0.125 and r_PMDA/MA_ = 0.125) even showed Shore A values higher than 80. Commercially available epoxy resins exhibited Shore D values around 80 [21]. However, all samples exhibited Shore D values lower than 40. Therefore, the composition of the epoxy resin as well as of the curing agents has to be improved. Kuncho et al. [15] also synthesized an epoxy material composed of ELO and MTHPA with a Shore D hardness of 87. On the one hand, the high level of hardness probably results from the curing for 1 h at 90 °C, followed by 8 h at 180 °C, whereby the cross-linking is complete, and on the other hand, of the different composition of the epoxy material and the usage of the catalyst 1,8-diazabicyclo[5.4.0]-undec-7-ene. With the decreasing molar ratio r_PMDA/MA,_ the Shore A and D hardness of the samples synthesized with a molar ratio r_MTHPA/MA_ of 1.000, 0.500 and 0.250 decreased as well. In the case of the samples prepared with a molar ratio r_MTHPA/MA_ of 0.125, the hardness decreased, while changing the molar ratio r_PMDA/MA_ of 1.000 to 0.500 and rising below 0.500.

### 3.2. Dependence of the Mass of ELO on the Properties of the Prepared Epoxy Resins

The PD418 sample was further investigated due to its promising results in terms of its short gel time (1.6 min) and its liquid state. The dependence of the ELO mass on the gel time was analyzed with different ELO masses (8, 10, 12, 14 and 16 g, Table 1). With increasing ELO mass, an increase in gel time was observed. From 8 to 14 g, gel time increased linearly from 1.0 to 2.1 min, while from 14 to 16 g, the increase was from 2.1 to 3.6 min. Shore A values of the samples were constant at around 80 until 12 g of ELO and decreased to 32 with higher ELO masses. A similar observation was made of the Shore D values. At a range of 8 to 12 g ELO, the Shore D remained constant at 21. At a utilized mass of 14 g ELO, a Shore D hardness of 13 was determined. For the sample prepared with 16 g ELO (PD418-16), Shore D could not be measured since the value was below the detectable value of 10. The results suggest that with increasing ELO mass, the process of cross-linking of ELO with the hardener is not completed due to insufficient hardener mass, simultaneously increasing the gel time and decreasing hardness [22]. As mentioned above, a balanced reaction equation is not feasible with this hardener system. 

### 3.3. Substitution of PMDA Using Biogenic Carboxylic Acids

**Figure 4:**
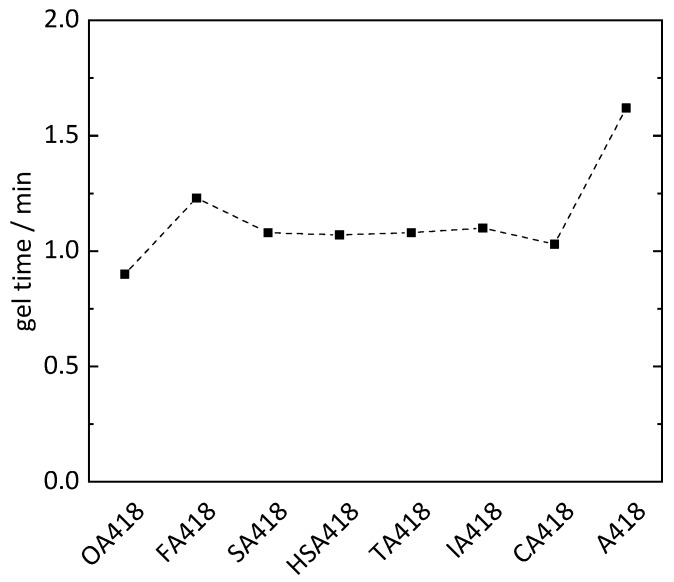
Gel time of epoxy resins utilizing different biogenic compounds (oxalic acid OA, fumaric acid FA, succinic acid SA, malic acid HSA, tartaric acid TA, itaconic acid IA and citric acid CA) in the hardener system (MA, MTHPA and X; X = biogenic compound).

To reduce the petrol-based chemicals in the epoxy resins, PMDA was substituted with oxalic acid (OA), fumaric acid (FA), succinic acid (SA), malic acid (HSA), tartaric acid (TA), itaconic acid (IA) and citric acid (CA) within the hardener system of sample PD418 (Table 2). On average, all samples cured with hardeners containing biogenic substrates showed shorter gel times (1.1 min) than sample PD418 (1.6 min). PMDA is a double carboxylic acid anhydride, whereby one molecule can form four possible bindings with ELO. In contrast, most of the biogenic compounds (OA, FA, SA and IA) are difunctional and can only form up to two bindings to ELO using the respective reactive hydrogen atoms. The shorter gel times can be explained by the more flexible structure due to incorporation of the biogenic compounds compared to the rigid molecule structure of PMDA. The shortest gel times (0.9 and 1.0 min) were exhibited by the OA418 and CA418 samples, respectively (Figure 4). CA is a tribasic acid possessing an additional alcohol functionality, whereby four protonic groups are present. Through these four reactive hydrogen atoms and the flexibility in its structure, cross-linking with the epoxy groups could be faster than cross-linking with hardener containing less reactive hydrogen atoms. The OA418 sample showed the shortest gel time of 0.9 min, although OA only has two reactive hydrogen atoms. Given the neighboring position of the carboxyl groups, the hydrogen atoms are more reactive than in the other substances, whereby cross-linking is presumably accelerated. However, a shorter gel time would be expected from the TA418 sample due to four reactive hydrogen atoms from the two carboxylic acids and two alcohol groups of the TA. However, the TA418 sample showed the same gel time as the FA418, SA418 and IA418 samples (1.1 min). The reactivity of the hydrogen atoms of the alcohol groups was possibly too weak compared to that of the carboxylic acids, whereby the gel process was not accelerated. 

**Figure 5:**
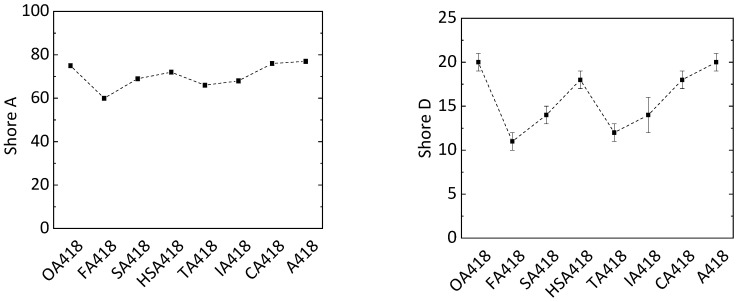
Shore A (**left**) and Shore D (**right**) hardness of epoxy resins with different biogenic compounds (oxalic acid OA, fumaric acid FA, succinic acid SA, malic acid HSA, tartaric acid TA, itaconic acid IA and citric acid CA) in the hardener system (MA, MTHPA and X; X = biogenic compound).

Shore A and D hardness of these samples was determined. The PD418 sample had a Shore A hardness of 77. The same hardness was also detected in samples OA418 (value of 75) and CA418 (value of 76) (Figure 5). The samples synthesized with other substrates had Shore A values around 15% lower (value of 67). A similar observation was made in the Shore D analysis. The Shore D hardness of the PD418 sample (value of 20) was similar to that of OA418 (value of 20) and CA418 (value of 18) when applying PMDA. Due to its rigid structure, it was assumed that the PD418 sample would show the highest hardness out of all samples. In the case of the OA418 sample, OA has the shortest chain length among the applied biogenic compounds, whereby the flexibility of the cross-linked system is probably lower than that of the cross-linked systems composed of FA, SA, HSA, TA and IA. Concerning the CA418 sample, through its three carboxylic groups and the alcohol group, CA has four reactive hydrogen atoms. By forming four possible bindings, the network is more cross-linked compared to those formed by FA, SA, HSA TA and IA. Since OA is harmful to health, CA is a more promising alternative to PMDA.

## 4. Conclusions

In this work, the effect of varying amounts of MTHPA, PMDA and MA in the hardener system upon the gel time of ELO was studied. Gel time tests at 80 °C showed that with decreasing molar ratios r_PMDA/MA_ and r_MTHPA/MA_ from 1.000 to 0.125, curing time decreases from 12.9 to 0.9 min. The hardness of the synthesized epoxy materials showed Shore A hardness over 80 and is classified as very hard. In contrast, values of Shore D hardness, which increased with higher molar ratios r_PMDA/MA_, were all under 40.

Curing of ELO with MTHPA, MA and biogenic multifunctional carboxylic acid compounds (OA, FA, SA, HSA, TA, IA or CA) resulted in novel bio-based epoxy materials showing shorter gel times and similar Shore A and Shore D hardness compared to an epoxy resin based on PDMA (referred to sample PD418). These effects were most significant in the case of epoxy resins cured with CA and OA, which showed the shortest gel times (1.0 min and 0.9 min, respectively) and the same Shore A and Shore D hardness of 76 and 20, respectively, as PD418. Therefore, citric acid is a promising non-toxic alternative for chemicals obtained from fossil fuels as a curing reagent. To synthesize epoxy materials with higher values of the Shore D hardness, the curing should take place at higher temperatures. Through substitution of PMDA, epoxy resins with a biogenic character of 77 wt % in the final mixture were produced. Further substitution of MA with biogenic acids will lead to epoxy materials with an even higher biogenic character. 

## Figures and Tables

**Table 1:** Chemical amounts of methyltetrahydrophthalic anhydride (MTHPA) n_MTHPA_, pyromellitic dianhydride (PMDA) n_PMDA_, maleic acid (MA) n_MA_ and mass of epoxidized linseed oil (ELO) m_ELO_ of the precursors.

Sample Name	n_MTHPA_/mmol	n_PMDA_/mmol	n_MA_/mmol	m_ELO_/g
PD111	8.0	8.0	8.0	12
PD212	10.2	5.1	10.3	12
PD414	11.9	3.0	11.9	12
PD818	12.9	1.6	12.9	12
PD122	4.8	9.6	9.6	12
PD112	6.5	6.5	13.0	12
PD214	7.9	3.9	15.8	12
PD418	8.9	2.2	17.7	12
PD144	2.7	10.6	10.7	12
PD124	3.7	7.5	15.0	12
PD114	4.7	4.7	18.9	12
PD218	5.4	2.7	21.6	12
PD188	1.4	11.3	11.3	12
PD148	2.1	8.1	16.3	12
PD128	2.6	5.2	20.9	12
PD118	3.1	3.0	24.4	12
PD418-ELO8	8.9	2.2	17.7	8
PD418-ELO10	8.9	2.2	17.7	10
PD418-ELO14	8.9	2.2	17.7	14
PD418-ELO16	8.9	2.2	17.7	16

**Table 2:** Chemical amounts of methyltetrahydrophthalic anhydride (MTHPA) n_MTHPA_, X^1^ n_X_^1^ and maleic acid (MA) n_MA_, and mass of epoxidized linseed oil (ELO) m_ELO_ of the precursors.

Sample Name	n_MTHPA_/mmol	n_X_^1^/mmol	n_MA_/mmol	m_ELO_/g
OA418	9.3	2.3	18.6	12
FA418	9.4	2.3	18.7	12
SA418	9.3	2.4	18.7	12
HSA418	9.3	2.3	18.5	12
TA418	9.2	2.3	18.4	12
IA418	9.3	2.3	18.6	12
CA418	8.9	2.2	17.8	12

X^1^ = OA, FA, SA, HSA, TA, IA or CA.
